# Delayed first active-phase meal, a breakfast-skipping model, led to increased body weight and shifted the circadian oscillation of the hepatic clock and lipid metabolism-related genes in rats fed a high-fat diet

**DOI:** 10.1371/journal.pone.0206669

**Published:** 2018-10-31

**Authors:** Hatsumi Shimizu, Fumiaki Hanzawa, Daeun Kim, Shumin Sun, Thomas Laurent, Miki Umeki, Saiko Ikeda, Satoshi Mochizuki, Hiroaki Oda

**Affiliations:** 1 Laboratory of Nutritional Biochemistry, Nagoya University, Nagoya, Japan; 2 Department of Nutritional Sciences, Nagoya University of Arts and Sciences, Nisshin, Japan; 3 Institute of Innovation for Future Society, Nagoya University, Nagoya, Japan; 4 Faculty of Education, Oita University, Oita, Japan; University of Lübeck, GERMANY

## Abstract

The circadian clock is closely related to human health, such as metabolic syndrome and cardiovascular disease. Our previous study revealed that irregular feeding induced abnormal lipid metabolism with disruption of the hepatic circadian clock. We hypothesized that breakfast skipping induces lipid abnormalities, such as adiposity, by altering the hepatic circadian oscillation of clock and lipid metabolism-related genes. Here, we established a delayed first active-phase meal (DFAM) protocol as a breakfast-skipping model. Briefly, rats were fed a high-fat diet during zeitgeber time (ZT) 12–24 in a control group and ZT 16–4 in the DFAM group. The DFAM group showed increased body weight gain and perirenal adipose tissue weight without a change in total food intake. The circadian oscillations of hepatic clock and *de novo* fatty acid synthesis genes were delayed by 2–4 h because of DFAM. The peaks of serum insulin, a synchronizer for the liver clock, bile acids, and non-esterified fatty acid (NEFA) were delayed by 4–6 h because of DFAM. Moreover, DFAM delayed the surge in body temperature by 4 h and may have contributed to the increase in body weight gain and adipose tissue weight because of decreased energy expenditure. These data indicated a potential molecular mechanism by which breakfast skipping induces abnormal lipid metabolism, which is related to the altered circadian oscillation of hepatic gene expression. The results also suggested that the delayed peaks of serum NEFA, bile acids, and insulin entrain the circadian rhythm of hepatic clock and lipid metabolism-related genes.

## Introduction

In mammals, physiological and behavior rhythms such as sleep-wake cycles, the endocrine system, body temperature, and locomotor activity are driven by circadian clocks [1,2]. Mammalian circadian clocks are organized as a hierarchical oscillator system [3]. The master clock, the suprachiasmatic nucleus (SCN), is located in the hypothalamus and is responsible for orchestrating peripheral clocks in the liver and other organs [4]. The circadian clock is formed by a negative feedback system [5]. The circadian locomotor output cycles protein kaput (CLOCK) and brain and muscle Arnt-like protein 1 (BMAL1) heterodimer activates the transcription of period (PER) and cryptochrome (CRY) genes. The PER and CRY proteins form a heterodimer that suppresses the transactivation by CLOCK/BMAL1 [5]. Other clock genes such as differentiated embryo chondrocytes (DEC), nuclear receptor subfamily 1, group D, member 1 (REV-ERBα), nuclear receptor subfamily 1, group D, member 2 (REV-ERBβ), and nuclear receptor subfamily 1, group F, member 1 (RORα) also participate in the negative feedback loop. Furthermore, several clock genes regulate carbohydrate, lipid, and amino acid metabolism [6–11].

In modern society, many people are exposed to irregular eating patterns such as shift work and jet lag. Recent studies have demonstrated a close relationship between eating behavior and several metabolic diseases [12–14]. Many studies have reported that females who work at irregular times, such as nurses and flight attendants, have a higher risk of cancer [15,16]; moreover, shift workers more frequently suffer health problems such as metabolic syndromes [17], cardiovascular diseases [18], cancer [19], and abnormal blood lipids [20]. Misalignment of diurnal oscillations of some hormones was suggested to contribute to a higher risk of diseases in shift workers [21]. Diurnal oscillations of insulin and glucagon are mainly controlled by feeding-fasting [22]. Additionally, oscillations of glucocorticoids are controlled by the circadian clock and feeding-fasting [23,24]. *Clock* mutant mice exhibited altered diurnal feeding rhythms and obesity [25]. *Bmal1*-knockout mice showed abnormal lipid metabolism [26]. Our previous study showed that irregular feeding induced hypercholesterolemia by disrupting the circadian oscillations of several clock genes and shifted the peak of the circadian oscillation of cholesterol 7 alpha-hydroxylase (CYP7A1) [27]. CYP7A1 is a rate-limiting enzyme in the reaction that converts cholesterol to bile acids, and its circadian oscillation is regulated by D site of albumin promoter binding protein (DBP), a liver-enriched transcriptional activator [28]. It has also been reported that time-restricted feeding ameliorates abnormal lipid metabolism and obesity induced by feeding of a high-fat diet in mice [29]. Time-restricted feeding of excess sucrose also ameliorated the development of fatty liver and hyperlipidemia in rats [30]. These studies indicate that feeding timing is important for health by changing the circadian oscillations of clock and nutrient metabolism-related genes.

Breakfast skipping has serious implications for human health. Breakfast is recognized as the most important meal among the commonly found 3-meals by day pattern, and many studies have reported the health benefits associated with breakfast [31,32], but not with lunch and dinner. In the USA and Europe, 10–30% of children and adolescents regularly skip breakfast [33]. In Japan, 28.2% of 20–29-year-olds were found to skip breakfast [34]. Many epidemiological studies have reported that breakfast skipping is associated with various health issues, such as a higher body mass index [35], and higher risks of metabolic syndrome [36], type 2 diabetes [37], and coronary heart disease [38]. Additionally, some studies have linked breakfast skipping with the learning performance of children and adolescents [39,40]. However, these studies were observational and did not determine the molecular mechanism underlying how breakfast skipping induces abnormal metabolic problems in humans. Several studies have investigated the effect of meal shifting on lipid metabolism and metabolic abnormalities in rodents [29,30,41–44]. Delayed meal timing for 6 h in active-phase induced heavier body weight, increased lipid synthesis, and delayed hepatic clock gene expression [41,42]. However, 2 h of delayed time-restricted feeding for 8 h in active phase ameliorated obesity induced by feeding a high-fat diet [29]. These studies indicated that the delayed period and duration of feeding are very important in lipid metabolism and clock gene regulation. To investigate the effect of breakfast skipping in an animal model, we set the duration of the feeding and delayed periods to 12 and 4 h, respectively, to more closely reflect human breakfast skipping compared to previous studies [29,41,42]. We hypothesized that breakfast skipping induces metabolic disorders through abnormal circadian oscillations. We established a breakfast-skipping rat model and investigated whether a 4-h delayed first active-phase meal (DFAM) affects lipid metabolism by altering the circadian oscillations of clock or lipid metabolism-related genes in the liver. We found that DFAM increased body weight gain and adipose tissue weight without changing the total food intake. We also found that peaks in the circadian oscillation of the hepatic clock and lipid metabolism-related genes were delayed by 2–4 h by DFAM. Thus, DFAM induced metabolic disorders by delaying the circadian oscillations of clock and lipid metabolism-related genes.

## Materials and methods

### Animals

The animal study was approved by the Animal Care Committee of Nagoya University (approval no.2015092501) and performed in compliance with the Rules and Regulations of the Guide for the Care and Use of Laboratory Animals, Nagoya University. Surgical procedures were performed under isoflurane anesthesia, and efforts were made to minimize suffering. Fifty-six 5-week-old male Wistar rats were purchased from Japan SLC (Shizuoka, Japan). Rats were housed in individual wire-bottomed cages and kept under a 12-h light cycle, zeitgeber time (ZT) 0–12. During the experimental periods, the rats were allowed free access to water. Rats were first provided a stock diet (Lab MR Breeder, Nosan Co., Yokohama, Japan) for 1 day and then a basal diet for 2 days before surgery. The composition of the basal diet was (in g/kg) sucrose, 218; starch, 435; corn oil, 50; casein, 200; cellulose, 50; vitamin mixture (AIN93-VX), 10; mineral mixture (AIN93-MX), 35; and choline chloride, 2. On day 3, the eight rats in the control group and nine rats in the DFAM group were implanted with a temperature data logger (KN Laboratories Osaka, Japan) into the intraperitoneal cavity. After surgery, the rats were allowed to recover from surgery for 3 days and fed a high-fat diet. The composition of the high-fat diet was (in g/kg) sucrose, 161; starch, 322; corn oil, 20; lard, 150; casein, 250; cellulose, 50; vitamin mixture (AIN93-VX), 10; mineral mixture (AIN93-MX), 35; and choline chloride, 2. The energy sources were 43.3% carbohydrate, 22.4% protein, and 34.3% lipid. The amount of lipid in the high-fat diet was determined based on the median lipid energy intake of the human population in the USA reported in 2005 [45]. Six days after the rats arrived, they were divided into control (n = 28) and DFAM groups (n = 28). Control rats were allowed access to a diet from ZT 12–24. The DFAM group was allowed access to a diet from ZT 16–4. We set the experiment to a fixed-duration of feeding time at 12 h, as previous studies showed that this duration affects lipid metabolism [29]. Although this DFAM protocol shifted the feeding time to a 4-h delay, we use this as a model of breakfast skipping. The daily food amount was calculated based on the amount of food intake on the previous day. The total amount of food was approximately 1.1-fold of that on the previous day. Therefore, the rats were provided enough food each day. Rats are nocturnal animals and eat approximately 80% of their total food intake during the dark period (ZT 12–24) [46]. The control group was fed one-third of the total daily food amount during ZT 12–16 and fed two-thirds during ZT 16–24. The DFAM group was fed two-thirds of a total daily food amount during ZT 16–24 and fed one-third during ZT 24–4. After their food was removed at ZT 0 for the control group and ZT 4 for the DFAM group, weighted, and total daily food intake was then calculated. We next calculated the amount of the next meal. The experimental period was 14–15 days. Four rats from each group were sacrificed at the 4-h intervals from day 14 ZT 2 to day 15 ZT 2. Their livers and epididymal adipose tissues were harvested and immediately frozen in liquid nitrogen. Subsequently, the tissues were stored at -80°C until use analysis.

### Measurement of body temperature

The body temperature of rats was measured using a temperature data logger, which was implanted into the intraperitoneal cavity as described above. Rats were allowed 3 days to recover from surgery. Body temperatures were recorded continuously during the experimental period every 10 min with a resolution of 0.1°C. The collected data were analyzed using the Rh Manager program (KN Laboratories Inc., Osaka, Japan).

### Biochemical analysis

After liver homogenization, hepatic lipids were extracted as described by Folch et al. [47]. The amount of total liver lipids was determined gravimetrically. The levels of hepatic lipids (cholesterol, triglyceride, phospholipids) and serum parameters (glucose, total cholesterol, triglyceride, non-esterified fatty acid (NEFA), total bile acids) were enzymatically determined using commercial kits (T-CHO and TG-EN; Kainos Laboratories, Tokyo, Japan; Phospholipids C-Test, Glucose CII-test, Cholesterol C-test, Triglyceride E-test, NEFA C-test and TBA-test; Wako Pure Chemical Industries, Osaka, Japan). Insulin and corticosterone were measured using commercial enzyme-linked immunosorbent assay (ELISA) kits (Rat insulin ELISA kit; Morinaga Institute of Biological Science, Yokohama, Japan; Corticosterone ELISA kit; Assaypro, St. Charles, MO, USA).

### Total RNA extraction and real-time quantitative PCR

Total RNA from the liver and epididymal adipose tissue of rats was extracted as described by Chomczynski and Sacchi [48]. The quality of RNA was confirmed by northern blotting. cDNA was synthesized using a total RNA by a High Capacity cDNA Reverse Transcription Kit (Applied Biosystems, Foster City, CA, USA). Real-time quantitative polymerase chain reaction (real-time PCR) was performed using 2X Power SYBR Master Mix (Applied Biosystems, Foster City, CA, USA) and analyzed with StepOnePlus (Applied Biosystems, Foster City, CA, USA). Primer sequences are shown in S1 Table. The relative levels of mRNA were normalized to those of Apolipoprotein E (Apo E).

### Rhythmicity analysis

The rhythmicity of circadian oscillation of body temperatures, serum parameters, clock genes, and lipid and glucose metabolism-related genes was analyzed by using JTK_CYCLE software [49]. JTK_CYCLE software implemented on R analyzed peak time (ZT) and amplitude of the rhythm under a 24-h period [49]. The rhythmicity was evaluated using a *p* < 0.05.

### Statistical analysis

The results were expressed as the mean ± standard error of the mean (SEM). The food intake, body weight gain, liver weight, and adipose tissue weight of both groups were analyzed by Student's *t*-test. The data of serum parameters and hepatic genes expression were analyzed by two-way analysis of variance (ANOVA). Statistical analysis was performed using IBM SPSS Statistics Version 22 software (SPSS, Inc., Chicago, IL, USA).

## Results

### DFAM increased body weight gain

**Fig 1:**
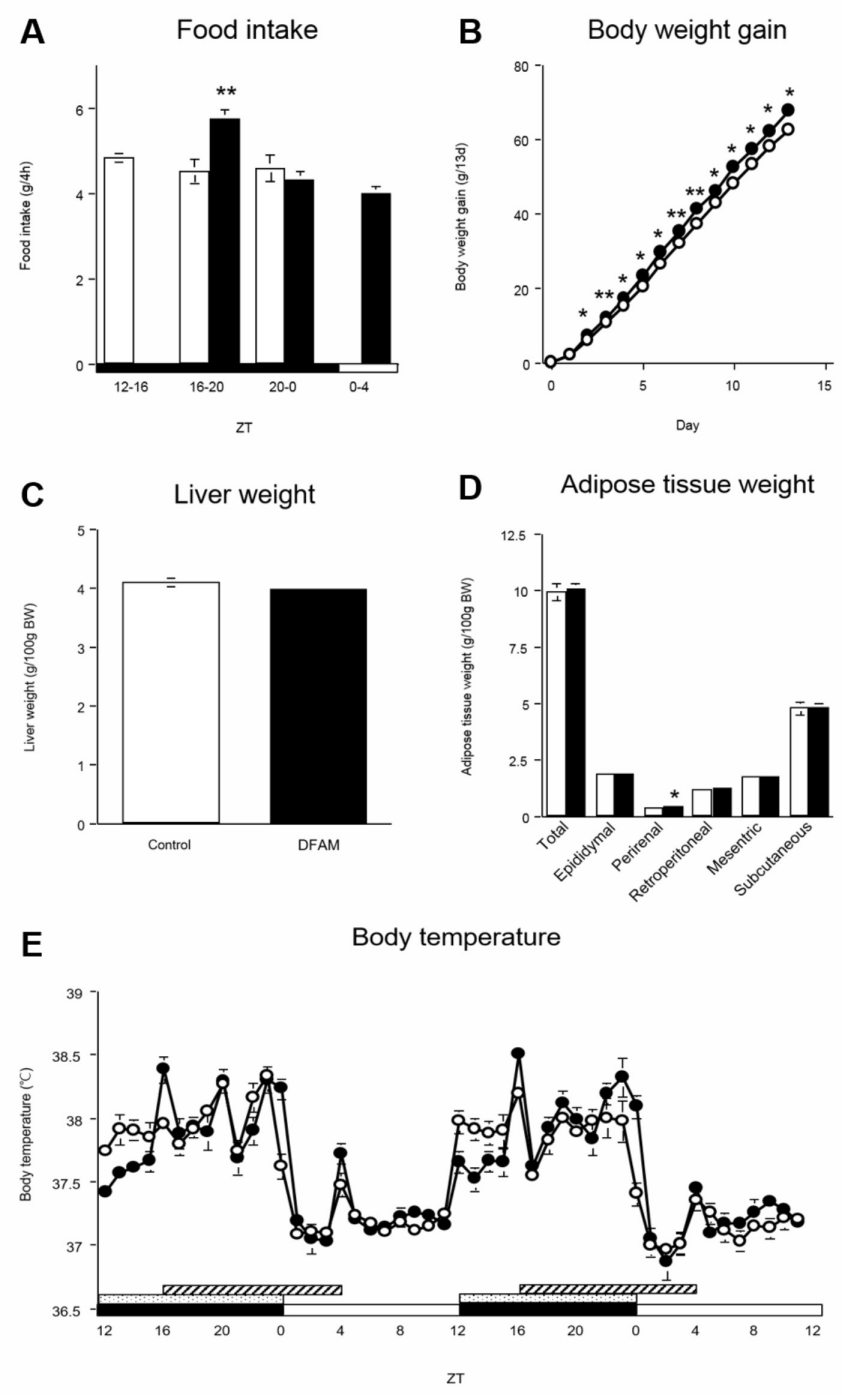
The body weight gain and perirenal adipose tissue were increased by DFAM. The open circle indicates control group (○) and the closed circle indicates the delayed the first active-phase meal (DFAM) group (●). The values are means ± SEM of 28 rats. (A) Food intake was measured by 4 times at intervals of 4 hours for a day. (B) Body weight gain of control and DFAM group rats was measured for 13 days. The DFAM group had showed more increased body weight gain than that of control group since the second day. (C) Liver was harvested by 7 times at intervals of 4 hours on 14 days and the weight of liver was measured at that time. (D) Five kinds of adipose tissue (epididymal adipose tissue, perirenal adipose tissue, retroperitoneal adipose tissue, mesenteric adipose tissue and subcutaneous adipose tissue) were also harvested by 7 times at intervals of 4 hours on 14 days and each weight of adipose tissue was measured at that time. Statistical significance of difference between values of food intake, body weight gain, liver weight and adipose tissue were analyzed by Student's *t-*test, respectively. The *, ** and *** are valued significantly difference (*p* <0.05, *p* <0.01 and *p* <0.001) from the control group by Student’s *t*-test. (E) Rats were implanted thermometer into intraperitoneal and thermometer were removed after experimental period (day 14). The data of body temperature were analyzed by 60 mins intervals for 2 days. The open and closed horizontal bars indicate the light (ZT 0–12) and dark (ZT 12–24), respectively. The dot and deviant crease line horizontal bars indicate the time of feeding, providing to control and DFAM group, respectively. The body temperature values are means ± SEM, n = 8 (control group) or 9 (DFAM group). The rhythmicity of body temperature was analyzed by JTK_CYCLE and the results were provided in S3 Table.

Total food intake during the experimental period was not significantly different between groups (control group: 168.3 ± 1.80 g/14 days; DFAM group: 175.4 ± 1.79 g/14 days, *p* > 0.05). Although DFAM rats consumed more food than control rats during ZT 16–20 (Fig 1A), DFAM rats consumed approximately 30% of the daily intake during ZT 0–4. Both groups consumed approximately 30% of the daily food every 4 h during the 12-h feeding period, as expected. Because both groups consumed similar amounts of the diet for 12 h, we predicted that the DFAM protocol could be applicable as a model for breakfast skipping.

The DFAM group showed increased body weight gain starting on day 2 (Fig 1B). The liver weight and hepatic lipid levels, including triglycerides, cholesterol, and phospholipids, were not different between groups (Fig 1C and S2 Table). However, total adipose tissue weight, which included epididymal, retroperitoneal, mesenteric, and subcutaneous adipose tissues, tended to increase with DFAM; perirenal adipose tissue weight was also significantly increased in the DFAM group compared to in the control group (Fig 1D). These results indicate that DFAM induced a higher body weight because of fat accumulation in the adipose tissue but not in the liver.

### DFAM delayed the surge in body temperature

The master regulator of circadian oscillation is the SCN. Additionally, the regulator of body temperature is located in the anterior hypothalamus, where circadian oscillation is regulated by the SCN [50]. In the control group, body temperature was elevated at the onset of the active phase (ZT 12) and decreased at the onset of the rest period (ZT 24). However, in the DFAM group, body temperature moderately increased at ZT 12, sharply increased at the time of eating (ZT 16), and then decreased at ZT 1, even if the rats were still consuming the diet until ZT 4 (Fig 1E). Locomotor activity is also an important factor to regulate the body temperature. We did not measure the locomotor activity and pattern in the present study. However, in another DFAM experiment using a similar experimental procedure, we found that the total and pattern of locomotor activity was similar in both the control and DFAM groups (S1 Fig). These data indicate that the surge in body temperature was regulated by both light and feeding, but the decrease in body temperature was regulated mainly by light.

### DFAM delayed peaks of serum insulin, NEFA, and bile acids

**Fig 2:**
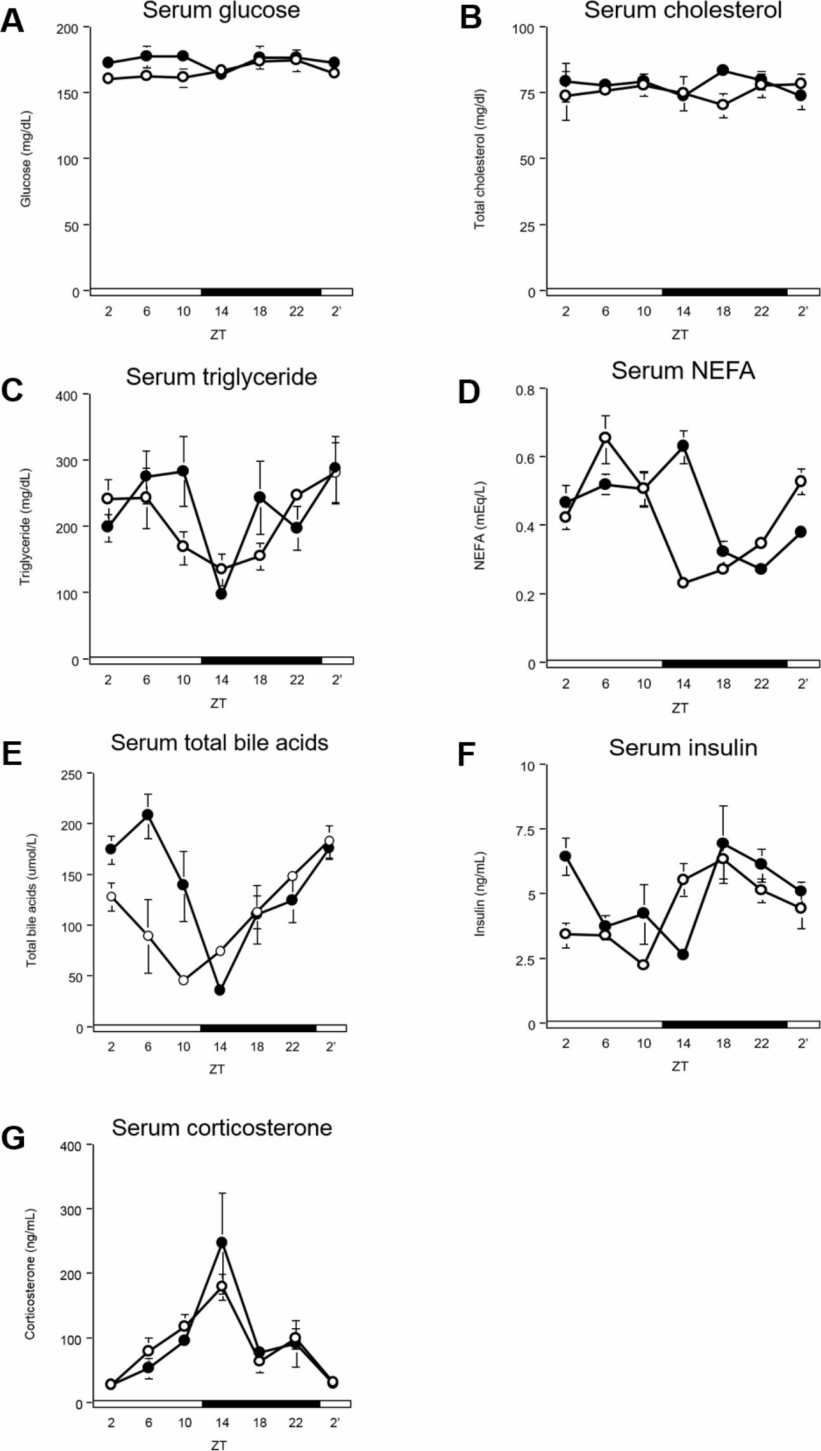
DFAM induced phase delay on NEFA, bile acids and insulin of serum in rat. We measured concentrations of (A) glucose, (B) cholesterol, (C) triglyceride, (D) NEFA, (E) total bile acids, (F) insulin, and (G) corticosterone in serum. The serum was harvested by 7 times at intervals of 4 hours from rats of control and DFAM group on day14. The open circle indicates control group (○) and the closed circle indicates DFAM group (●). Each values of concentration is mean ± SEM of 4 rats. The open and closed horizontal bars indicate the light (ZT 0–12) and dark (ZT 12–24), respectively. The rhythmicity of serum parameter was analyzed by JTK_CYCLE and the results were provided in S3 Table. The statistical significance of difference was analyzed by two-way ANOVA. The results of two-way ANOVA were described in S7 Table.

Serum glucose, cholesterol, and triglyceride levels were not changed by DFAM (Fig 2A–2C and S3 Table). The peak of serum NEFA was delayed by 4 h from ZT 4 in the control group to ZT 8 in the DFAM group (Fig 2D and S3 Table). The peak of serum bile acids was delayed by 6 h from ZT 22 in the control group to ZT 4 in the DFAM group (Fig 2E and S3 Table). The peak of serum insulin was delayed by 4 h from ZT 18 in the control group to ZT 22 in the DFAM group (Fig 2F and S3 Table). The serum corticosterone level was not changed by DFAM (Fig 2G and S3 Table). These data suggest that DFAM delayed the circadian oscillations of NEFA, bile acids, and insulin, and these changes mediated the delayed circadian oscillation of clock and lipid metabolism-related genes (see below).

### DFAM delayed circadian oscillations of hepatic clock genes and lipid metabolism-related genes

**Fig 3:**
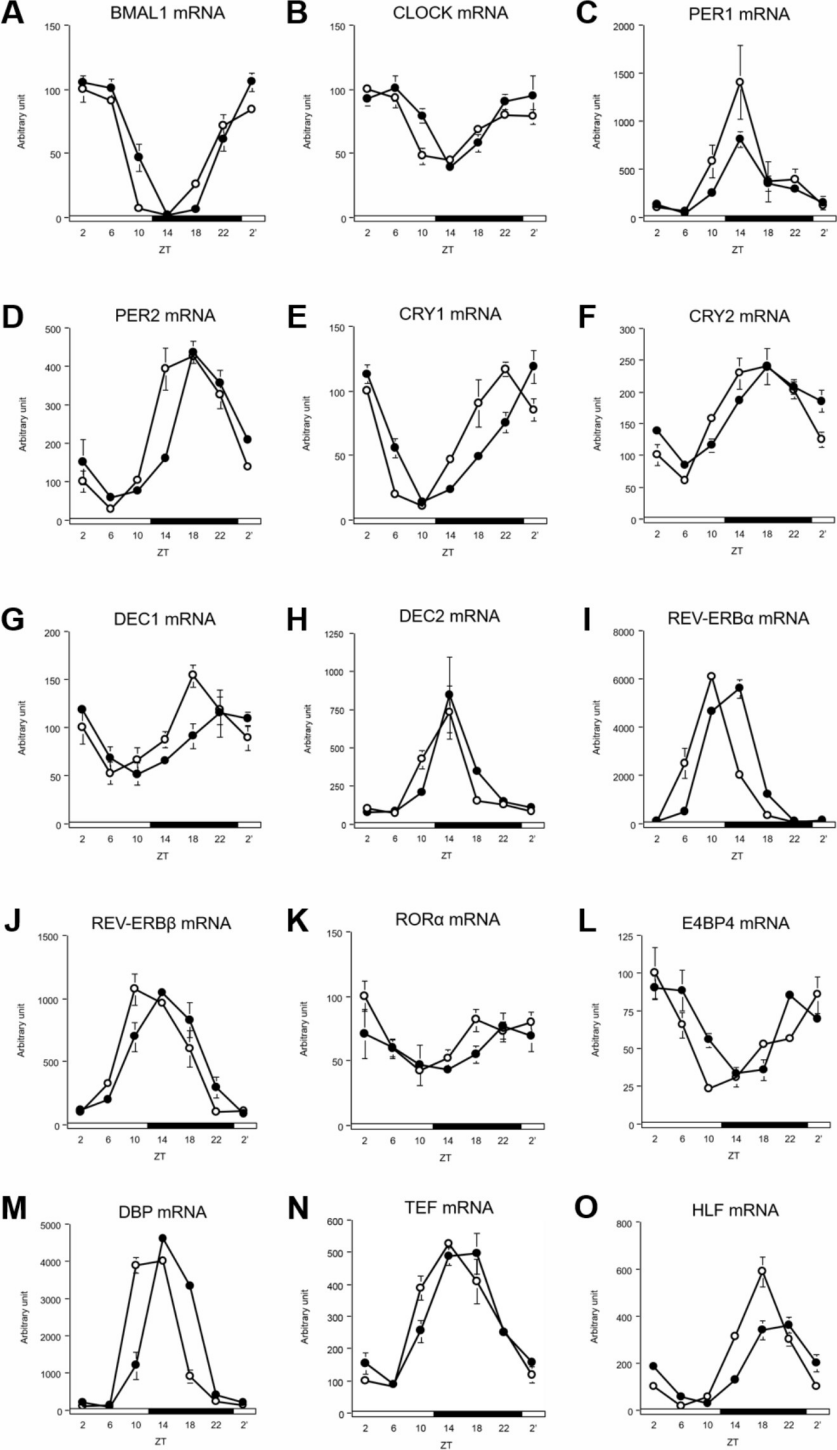
DFAM altered circadian oscillation of hepatic clock genes in rats. We profiled circadian oscillation of hepatic clock genes. We measured mRNA expression circadian oscillations of (A) BMAL1, (B) CLOCK, (C) PER1, (D) PER2, (E) CRY1, (F) CRY2, (G) DEC1, (H) DEC2, (I) REV-ERBα, (J) REV-ERBβ, (K) RORα, (L) E4BP4, (M) DBP, (N) TEF and (O) HLF genes related hepatic circadian clock. The mRNA expressions were analyzed using real-time PCR and those were normalized by Apo E gene expression levels. The open circle indicates control group (○) and the closed circle indicates DFAM group (●). Each value in ZT point is means ± SEM of 4 rats. The open and closed horizontal bars indicate the light (ZT 0–12) and dark (ZT 12–24), respectively. The rhythmicity of hepatic circadian clock gene expression was analyzed by JTK_CYCLE and the results were provided in S4 Table. The statistical significance of differences was analyzed by two-way ANOVA. The results of two-way ANOVA were described in S7 Table.

The peaks of CRY1, DEC1, and DBP mRNA were delayed by 4 h because of DFAM (Fig 3E, 3G and 3M and S4 Table). The peaks of BMAL1, CLOCK, PER1, CRY2, REV-ERBα, REV-ERBβ, thyrotrophic embryonic factor (TEF), and hepatic leukemia factor (HLF) mRNA were delayed by 2 h because of DFAM (Fig 3A–3C, 3F, 3I, 3J, 3N and 3O and S4 Table). In contrast, the peaks of PER2, DEC2, RORα, and E4BP4 mRNA were not changed by DFAM (Fig 3D, 3H, 3K and 3L and S4 Table). These data revealed that even a 4-h delay in the onset of eating can induce a 2–4-h delay in the circadian oscillation of liver clock genes.

**Fig 4:**
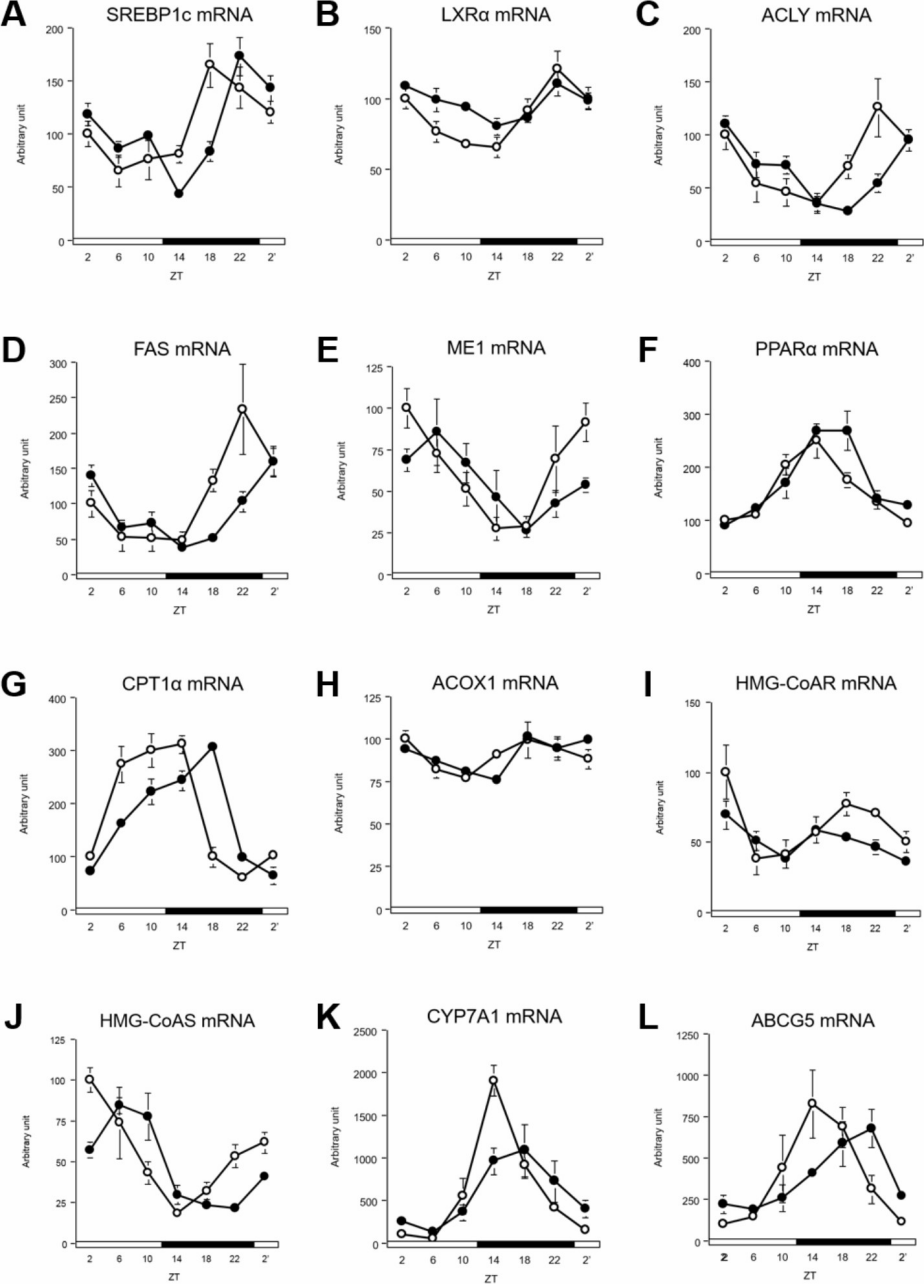
DFAM delayed circadian oscillation of lipid metabolism related genes in liver. We profiled circadian oscillations of lipid metabolism related genes in liver. We measure mRNA expression oscillations of (A) SREBP1c, (B) LXRα, (C) ACLY, (D) FAS, and (E) ME1 genes related with fatty acid synthesis, (F) PPARα, (G) CPT1α and (H) ACOX1 genes related with fatty acid degradation and (I) HMG-CoAR, (J) HMG-CoAS, (K) CYP7A1 and (L) ABCG5 genes related cholesterol metabolism. The mRNA expressions of hepatic lipid metabolism related genes were analyzed using real-time PCR and those were normalized by Apo E gene expression levels. The open circle indicates control group (○) and the closed circle indicates DFAM group (●). Each value in ZT point is means ± SEM of 4 rats. The open and closed horizontal bars indicate the light (ZT 0–12) and dark (ZT 12–24), respectively. The rhythmicity of hepatic lipid metabolism related gene expression was analyzed by JTK_CYCLE and the results were provided in S5 Table. The statistical significance of differences was analyzed by two-way ANOVA. The results of two-way ANOVA were described in S7 Table.

Two key transcription factors, sterol regulatory element-binding protein 1 (SREBP1c) for fatty acid synthesis and nuclear receptor subfamily 1, group C, member 1 (PPARα) for fatty acid β-oxidation, are known to be regulated by clock genes [51,52]. In this study, these genes showed a diurnal rhythm in the liver (Fig 4A and 4F and S5 Table). Nuclear receptor subfamily 1, group H, member 3 (LXRα), which transactivates SREBP1c [53], also showed a diurnal rhythm (Fig 4B and S5 Table). ATP citrate lyase (ACLY) and fatty acid synthase (FAS), which are regulated by SREBP1c, also showed a diurnal rhythm (Fig 4C and 4D and S5 Table) [54,55]. Malic enzyme 1 (ME1), which provides NADPH for lipogenesis [56], showed a diurnal rhythm as well (Fig 4E and S5 Table). Carnitine palmitoyltransferase 1A (CPT1α) and acyl-CoA oxidase 1 alpha (ACOX1), which are regulated by PPARα, showed significant oscillations (Fig 4G and 4H, and S5 Table) [57]. Fatty acid synthesis-related genes, such as SREBP1c and LXRα, showed mRNA peaks that were delayed by 2 h and ACLY, FAS, and ME1 showed mRNA peaks that were delayed by 4 h because of DFAM (Fig 4A–4E and S5 Table). Fatty acid β-oxidation-related genes, such as CPT1α and ACOX1, showed mRNA peaks that were delayed by 2–4 h because of DFAM; however, the peak of PPARα mRNA was not changed in both groups (Fig 4F–4H and S5 Table).

Cholesterol synthesis-related genes, such as 3-hydroxy-3-methylglutaryl-CoA reductase (HMG-CoAR), which codes for a rate-limiting enzyme during cholesterol synthesis, did not exhibit a diurnal rhythm in either group (Fig 4I and S5 Table). However, 3-hydroxy-3-methylglutaryl-CoA synthase 1 (HMG-CoAS) mRNA showed a diurnal rhythm, and its peak was delayed by 6 h because of DFAM (Fig 4J and S5 Table). Cholesterol degradation-related genes, such as CYP7A1, did not exhibit phase changes because of DFAM (Fig 4K and S5 Table). ATP-binding cassette subfamily G member 5 (ABCG5) mRNA showed a diurnal rhythm, and its peak was delayed by 2 h because of DFAM (Fig 4L and S5 Table). These data indicate that the peaks of fatty acid metabolism-related and cholesterol metabolism-related genes were delayed by 2–6 h because of DFAM.

### DFAM delayed circadian oscillations of glucose metabolism-related genes

**Fig 5:**
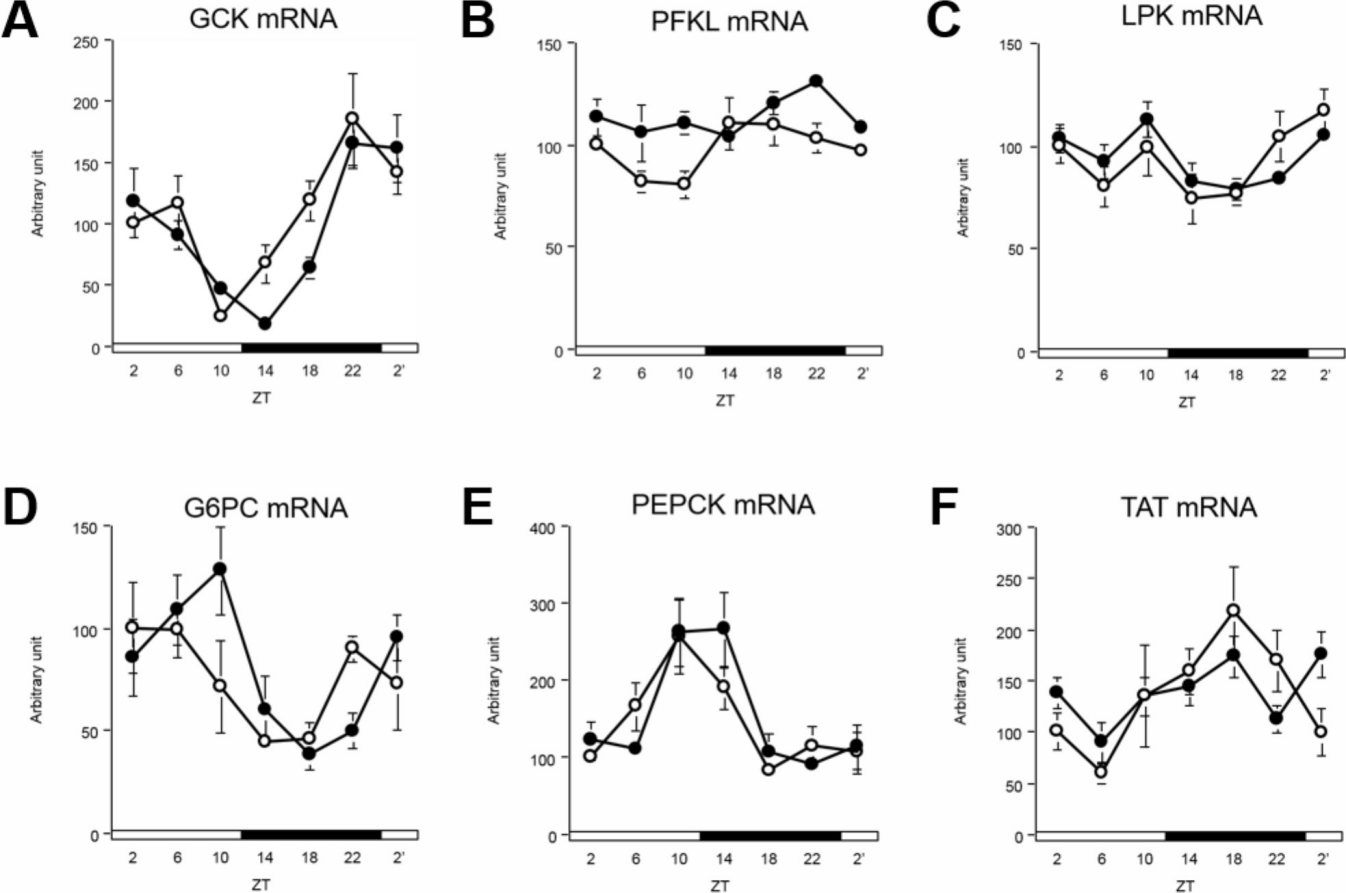
DFAM delayed circadian oscillation of glucose metabolism related genes in liver. We profiled expression of glucose metabolism related genes in liver. We measured circadian oscillation of mRNA of (A) GCK, (B) PFKL, (C) LPK, (D) G6PC, (E) PEPCK, and (F) TAT, genes related with glucose metabolism. The mRNA expressions of each gene were analyzed using real-time PCR and those were normalized by Apo E gene expression levels. The open circle indicates control group (○) and the closed circle indicates DFAM group (●). Each value in ZT point is means ± SEM of 4 rats. The open and closed horizontal bars indicate the light (ZT 0–12) and dark (ZT 12–24), respectively. The rhythmicity of hepatic glucose metabolism related gene expression was analyzed by JTK_CYCLE and the results were provided in S6 Table. The statistical significance of differences was analyzed by two-way ANOVA. The results of two-way ANOVA were described in S7 Table.

Glycolysis-related genes, such as glucokinase (GCK), showed diurnal rhythms, and their mRNA peak were delayed by 2 h because of breakfast skipping (Fig 5A and S6 Table). Phosphofructokinase, liver type (PFKL) and pyruvate kinase liver and red blood cell (LPK) mRNA did not show rhythmic changes (Fig 5B and 5C and S6 Table). Gluconeogenesis-related genes, including glucose-6-phosphatase, catalytic subunit (G6PC) and phosphoenolpyruvate carboxykinase 1 (PEPCK), mRNA showed diurnal rhythms, and their peaks were delayed by 2–4 h because of breakfast skipping (Fig 5D and 5E and S6 Table). However, tyrosine aminotransferase (TAT) mRNA did not show circadian oscillation with DFAM (Fig 5F and S6 Table). These data show that the peak of glucose metabolism-related genes was delayed by 2–4 h because of DFAM.

### DFAM slightly affected circadian oscillations of clock genes in epididymal adipose tissue

We also analyzed the circadian oscillation of clock genes in epididymal adipose tissue (S2 Fig, S8 Table and S9 Table). Unfortunately, we only analyzed epididymal adipose tissue, but not perirenal adipose tissue, because we stored only epididymal adipose tissues. The pattern of circadian oscillation of clock genes was very similar in several clock genes. Oscillations in PER1 and PER2 gene expression were delayed by DFAM (S2 Fig, S8 Table and S9 Table).

## Discussion

Breakfast skipping increases the risk of obesity, higher body mass index, metabolic syndrome, and type II diabetes in humans [35–38]. However, it is difficult to identify the molecular mechanism underlying how breakfast skipping induces metabolic disorders. In the present study, we hypothesized that breakfast skipping induces abnormalities in lipid metabolism by altering the hepatic circadian clock. To investigate the underlying molecular mechanism of the effect of breakfast skipping on metabolism, we developed a DFAM protocol. The rats showed significantly increased body weight gain and perirenal adipose tissue weight and exhibited a tendency for increased total adipose tissue weight upon undergoing DFAM (Fig 1B and 1D).

Circadian oscillations in the peripheral tissues, such as in the liver, are controlled by the SCN and feeding behavior [58]. In our previous study, we found that insulin synchronized the circadian clocks in the liver [59]. In the present study, peaks of hepatic clock genes were delayed by 2–4 h because of DFAM (Fig 3 and S4 Table). The delayed hepatic circadian clock oscillations may be mediated through the 4-h delay in the circadian oscillation of insulin (Fig 2F and S3 Table). Additionally, the circadian oscillations of serum NEFA and bile acids were also delayed by 4–6 h because of DFAM (Fig 2D and 2E and S3 Table). Although serum bile acid oscillation was delayed by DFAM, oscillation of the CYP7A1 gene was not affected. Enterohepatic circulation of bile acids may mainly control circadian oscillation. These data suggest that serum NEFA and bile acids function as synchronizers of the hepatic circadian clock. It has been reported that the amplitude of circadian oscillation was enhanced by adding bile acids to Caco2 cells; further the expression of hepatic circadian clock genes was significantly changed upon oral administration of bile acids in mice [60]. NEFA may enhance the entraining activity of insulin by accelerating insulin secretion from β cells by NEFA [61,62]; however, no studies have directly examined whether NEFA affects the circadian clock. NEFA and bile acids may function together to synchronize the circadian clock.

Numerous studies have indicated that disruption of the circadian oscillation of clock genes induces abnormal lipid metabolism [7,10,25–28,51]. *CLOCK* mutant mice exhibited abnormal diurnal feeding rhythm and obesity [25]. *BMAL1*-knockout mice showed suppressed adipogenesis [26]. Moreover, *REV-ERBα*-knockout mice showed abnormal blood lipid levels by altering hepatic circadian rhythms [51]. In our previous study, suppressing the feeding rhythm induced hypercholesterolemia by advancing the phase of CYP7A1 by altering the circadian oscillation of DBP in rats [27]. Because metabolic pathways are generally controlled systematically, once the active phases of regulatory enzymes are delayed or advanced by several hours, orchestrated metabolic regulation can be disrupted. In the present study, the peaks of lipogenesis-related genes were delayed by 2–4 h and those of cholesterol metabolism-related genes were delayed by 4–6 h because of DFAM (Fig 4 and S5 Table). These results suggest that changes in the circadian oscillation of clock and lipogenesis genes caused by DFAM contribute to abnormal lipid metabolism. Metabolic pathways, such as lipogenesis, glycolysis, and the pentose phosphate pathway, are interconnected. The circadian oscillation of glucose metabolism-related genes, such as GCK, was also delayed by 2–4 h because of breakfast skipping (Fig 5 and S6 Table). These results suggest that misalignment of the circadian oscillation of glycolysis contributes to abnormal lipid metabolism caused by DFAM.

The circadian oscillation of body temperature is controlled by a regulator in the anterior hypothalamus that shows circadian oscillations under control of the SCN [50]. Body temperature in control rats was elevated at ZT 12 and decreased at ZT 24 (Fig 1E). In the DFAM group, body temperature moderately increased at ZT 12 and sharply increased at ZT 16 (Fig 1E) when the rats began eating. This indicates that the surge in body temperature was regulated not only by light, but also by feeding. However, the decrease in body temperature of the rats in the DFAM group was only delayed by 1 h compared to that in the control group, even if the rats were still consuming the diet during ZT 0–4 (Fig 1A and Fig 1E). Locomotor activity is an important factor regulating the body temperature. Although we did not measure the locomotor activity and pattern in the present study, we found that the total and pattern of locomotor activity were similar in both the control and DFAM groups in another DFAM experiment (S1 Fig). Thus, we predicted that the surge in body temperature was regulated by both feeding and light, whereas the decrease in body temperature was mainly regulated by light. We estimated and compared the area of body temperature difference between both groups during ZT 12–16 and ZT 0–4. The area of ZT 12–16 was approximately 1.7-fold greater than that of ZT 0–4. These results suggest that reduced energy expenditure because of the 4-h delay in the body temperature surge led to increased body weight gain and accumulation of lipids in adipose tissues upon DFAM.

The present study revealed that DFAM increased body weight gain because of fat accumulation in the adipose tissues. Even with only a 4-h delay at the onset of the first active-phase meal, breakfast, the circadian oscillation of clock and lipid metabolism-related genes were delayed by 2–4 h. These results suggest that the delayed circadian rhythm of clock genes and lipid metabolism leads to increased body and adipose tissue weights. This study proposes that metabolic abnormality and obesity caused by breakfast skipping in humans would be mediated through alterations in the circadian rhythms of the peripheral tissues, such as the liver.

## Supporting information

S1 Table Primer sequences for quantitative real-time PCR. (PDF)

S2 Table The amount of hepatic lipids in DFAM rats. (PDF)

S3 Table The JTK_CYCLE analysis of body temperature and circadian fluctuations in serum parameters in DFAM rats (Related to Fig 1E and Fig 2). (PDF)

S4 Table The JTK_CYCLE analysis of circadian fluctuations in hepatic clock genes in DFAM rats (related to Fig 3). (PDF)

S5 Table The JTK_CYCLE analysis of circadian fluctuations in hepatic lipid metabolism related genes in DFAM rats (related to Fig 4). (PDF)

S6 Table The JTK_CYCLE analysis of circadian fluctuations in hepatic glucose metabolism related genes in DFAM rats (related to Fig 5). (PDF)

S7 Table The results of two-way ANOVA of in serum parameters, hepatic genes expression in DFAM rats. (PDF)

S8 Table The JTK_CYCLE analysis of circadian fluctuations in epididymal adipose tissue of DFAM rats (Related to S2 Fig). (PDF)

S9 Table The results of two-way ANOVA of gene expressions in epididymal adipose tissue of DFAM rats (Related to S2 Fig). (PDF)

S1 Fig DFAM did not change locomotor activity in rats fed a high-cholesterol diet. (PDF)

S2 Fig DFAM slightly delayed circadian oscillation of clock genes in epididymal adipose tissue. (PDF)
