# Supplementary material for: Delayed first active-phase meal, a breakfast-skipping model, led to increased body weight and shifted the circadian oscillation of the hepatic clock and lipid metabolism-related genes in rats fed a high-fat diet
Source: PLoS One. 2018 Oct 31;13(10):e0206669. doi: 10.1371/journal.pone.0206669 (PMC6209334; doi:10.1371/journal.pone.0206669)
Supplement: S1 Table — (PDF) [file pone.0206669.s001.pdf]

Supplementary Table 1. Primer sequences for quantitative real-time PCR.

Gene	Accession No.	Forward primer (5'→3')	Reverse Primer (5'→3')
ABCG5	NM_053754.2	TGTCCTTCAGCGTCAGCAAC	TCTGGCCACTCTCGATGTAC
ACLY	NM_016987.2	TGGACTTTGACTACGTGTGC	AAGACAGGGATCAGGATTTC
ACOX1	NM_017340.2	TGATGAAATACGCCCAGGTG	CCCATACGTCAGCTTGTTAC
APOE	NM_001270681.1	TGAACCGCTTCTGGGATTAC	TGGGTGACTTGGGAGCTCTG
BMAL1	NM_024362.2	TTTGTAGATCAGAGGGCGAC	GTACCTAGAAGTTCCTGTGG
CLOCK	NM_021856.2	AGTTAGGGCTGAAAGACGGC	TTCCAGTGCTTCCTTGAGAC
CPT1α	NM_031559.2	GGGATTAAGGTTCTGCTATG	AAGATGTGTGAGGAAGGTGG
CRY1	NM_198750.2	CACTATGCTCACGGAGACAG	CCCATGGAGCTTCTTCTTTG
CRY2	NM_133405.2	CTGGATAAGCACTTGGAACG	ACAGGCGGTAGTAGAAGAGG
CYP7A1	NM_012942.2	TGTGTGAGGGACCAGGTCTCT	AGCTCCAAAAGGTTGGAGGA
DBP	NM_012543.3	CTCTAGGGACACACCCAGTCCT	AGGCTTCAATTCCTCCTCTGAGA
DEC1	NM_053328.1	GAAACCATTGGACTCAGCTC	TTTCCCAGGGCCTTCTGATC
DEC2	XM_002729454.6	TAACAGCCTTAACGGAGCAG	CAAGACTTCTTTGGCGCAGG
E4BP4	NM_053727.2	ACTGGCATCACAAAGAACTG	ACTGGCATCACAAAGAACTG
FAS	NM_017332.1	CCAAGCAGGCACACACAATG	GATACCTCCGTCGACAATAG
G6PC	NM_013098.2	TCCGGTGCTTGAATGTCGTC	TCTGGAGGCTGGCATTGTAG
GCK	NM_001270849.1	AGAAGATCATCGGTGGGAAG	TTAAGCAGCACAAGTCGTAC
HLF	NM_024359.1	CGCTTTGCCTTCTGCTCATC	CCTGTGTAGGATGCTCTTTC
HMG-CoAR	NM_013134.2	TGCACAGACTCCTCAGACGTG	TTCGTCAAAACACCAGCTTCC
HMG-CoAS	NM_017268.1	ACACACATCACTTAGCCAAC	CCACTCCTTCATCCAAACTG
LXRα	NM_031527.2	TCCGAGATCTGGGATGTCCA	TGCCATGCAAGGGTCTCTTT
ME1	NM_012600.2	CTTGTTGCCACCCTGCATTG	TAAGCACACTGTACAACAGC
PEPCK	NM_198780.3	AACTGTTGGCTGGCTCTCAC	TCTGCTCTTGGGTAATGATG
PER1	NM_001034125.1	ACCAGCTCAAGGCTTAGGAGCT	TGGGATTTGGAGAGACCACTTC
PER2	NM_031678.1	CAACCTTTGTCTGCCATATGAGG	CGTTAGAAACACAAGCTCTTCCAC
PFKL	NM_01390.4	TGAGCATAGACAAGGGTTTC	CCGAGTTCCATGTGAGTTCC
LPK	NM_012624.3	CTGAGCGTATCCTGAAATGC	AAATAGGGTGTAACTGGGTC
PPARα	NM_013196.1	TTCGGCTAAAGCTGGCGTAC	ACTGGCATTTGTTCCGGTTC
REV-ERBα	NM_001113422.1	CCTTTGAGGTGCTGATGGTG	ACATGACTGTCTGGTCCTTC
REV-ERBβ	NM_147210.2	TGAACTTCTCCAGTGCTTAC	ACTTGCTCATAGGACAAACC
RORα	XM_008766408.2	ACGATGACCTCAGCACCTAC	CACATATGGGTTCGGGTTTG
SREBP1c	NM_001276708.1	GGAGCCATGGATTGCACATT	AGGAAGGCTTCCAGAGAGGA
TAT	NM_012668.2	CTACGTGATTCAGACGGATG	TCTTTCTTCCTTGTACCGAG
TEF	NM_019194.2	CCCTTGAGTATCAGCAGGTC	CAGTGTAATGTCTCCAGAGC
