# Supplementary material for: Delayed first active-phase meal, a breakfast-skipping model, led to increased body weight and shifted the circadian oscillation of the hepatic clock and lipid metabolism-related genes in rats fed a high-fat diet
Source: PLoS One. 2018 Oct 31;13(10):e0206669. doi: 10.1371/journal.pone.0206669 (PMC6209334; doi:10.1371/journal.pone.0206669)
Supplement: S2 Table — (PDF) [file pone.0206669.s002.pdf]

Supplementary Table 2. The amount of hepatic lipids in DFAM rats.

	Control	DFAM
	mg/g liver	
Total lipid	59.24 ± 0.81[ab]	56.84 ± 0.70
Triglyceride	20.58 ± 0.49	18.20 ± 0.32
Cholesterol	3.57 ± 0.05	3.45 ± 0.04
Phospholipid	24.24 ± 0.18	24.04 ± 0.18

[a] Values are means ± SEM, n=28.

[b] Statistical significance of difference between values were analyzed by Student's *t*-test. All data were not significant (p>0.05).
