# Supplementary material for: Delayed first active-phase meal, a breakfast-skipping model, led to increased body weight and shifted the circadian oscillation of the hepatic clock and lipid metabolism-related genes in rats fed a high-fat diet
Source: PLoS One. 2018 Oct 31;13(10):e0206669. doi: 10.1371/journal.pone.0206669 (PMC6209334; doi:10.1371/journal.pone.0206669)
Supplement: S3 Table — (PDF) [file pone.0206669.s003.pdf]

Supplementary Table 3. The JTK_CYCLE analysis of body temperature and circadian fluctuations in serum parameter in DFAM rats (Related to Fig 1E and Fig 2).

	Control			DFAM		
	p-value	Peak time (ZT)	Amplitude	*p*-value	Peak time (ZT)	Amplitude
Body temperature	0.000	24	0.566	0.000	24	0.519
Serum glucose	0.189	20	4.405	1.000	6	2.832
Serum cholesterol	1.000	22	2.132	1.000	10	2.132
Serum triglyceride	0.004	2	59.077	0.231	4	54.078
Serum NEFA	0.000	4	0.166	0.001	8	0.146
Serum total bile acids	0.000	22	49.646	0.001	4	63.187
Serum insulin	0.001	18	1.792	0.001	22	2.138
Serum corticosterone	0.000	12	35.401	0.000	12	44.749
