# Supplementary material for: Delayed first active-phase meal, a breakfast-skipping model, led to increased body weight and shifted the circadian oscillation of the hepatic clock and lipid metabolism-related genes in rats fed a high-fat diet
Source: PLoS One. 2018 Oct 31;13(10):e0206669. doi: 10.1371/journal.pone.0206669 (PMC6209334; doi:10.1371/journal.pone.0206669)
Supplement: S4 Table — (PDF) [file pone.0206669.s004.pdf]

Supplementary Table 4. The JTK_CYCLE analysis of circadian fluctuations in hepatic clock genes in DFAM rats (related to Fig 3).

	Control			DFAM		
Hepatic clock gene	*p*-value	Peak time (ZT)	Amplitude	*p*-value	Peak time (ZT)	Amplitude
BMAL1	0.000	0	49.466	0.000	2	45.857
CLOCK	0.002	0	21.287	0.003	2	20.631
PER1	0.001	14	19.079	0.000	16	15.913
PER2	0.000	18	54.247	0.000	18	37.859
CRY1	0.000	20	43.541	0.000	0	19.932
CRY2	0.000	16	83.835	0.000	18	68.529
DEC1	0.000	18	26.586	0.000	22	23.106
DEC2	0.000	14	157.344	0.000	14	138.799
REV-ERBα	0.000	10	34.233	0.000	12	46.731
REV-ERBβ	0.000	12	523.961	0.000	14	494.034
RORα	0.001	22	21.505	0.110	22	13.593
E4BP4	0.000	0	26.582	0.000	0	36.720
DBP	0.000	12	61.582	0.000	16	50.912
TEF	0.000	14	199.577	0.000	16	177.168
HLF	0.000	18	34.908	0.000	20	27.352
