# Supplementary material for: Delayed first active-phase meal, a breakfast-skipping model, led to increased body weight and shifted the circadian oscillation of the hepatic clock and lipid metabolism-related genes in rats fed a high-fat diet
Source: PLoS One. 2018 Oct 31;13(10):e0206669. doi: 10.1371/journal.pone.0206669 (PMC6209334; doi:10.1371/journal.pone.0206669)
Supplement: S5 Table — (PDF) [file pone.0206669.s005.pdf]

Supplementary Table 5. The JTK_CYCLE analysis of circadian fluctuations in hepatic lipid metabolism related genes in DFAM rats (related to Fig 4).

	Control			DFAM		
Hepatic lipid metabolism related gene	*p*-value	Peak time (ZT)	Amplitude	*p*-value	Peak time (ZT)	Amplitude
SREBP1c	0.001	20	27.552	0.000	22	28.380
LXRα	0.000	22	19.226	0.006	0	10.663
ACLY	0.000	22	33.628	0.001	2	16.157
FAS	0.000	20	29.501	0.000	0	12.403
ME1	0.000	2	33.592	0.005	6	27.337
PPARα	0.000	14	22.284	0.000	14	31.630
CPT1α	0.000	10	36.317	0.000	14	31.296
ACOX1	0.010	20	8.902	0.009	22	10.702
HMG-CoAR	0.077	20	11.964	0.098	20	17.918
HMG-CoAS	0.000	0	28.785	0.000	6	33.167
CYP7A1	0.000	16	28.714	0.000	16	19.812
ABCG5	0.000	14	38.620	0.000	18	25.003
