# Supplementary material for: Delayed first active-phase meal, a breakfast-skipping model, led to increased body weight and shifted the circadian oscillation of the hepatic clock and lipid metabolism-related genes in rats fed a high-fat diet
Source: PLoS One. 2018 Oct 31;13(10):e0206669. doi: 10.1371/journal.pone.0206669 (PMC6209334; doi:10.1371/journal.pone.0206669)
Supplement: S6 Table — (PDF) [file pone.0206669.s006.pdf]

Supplementary Table 6. The JTK_CYCLE analysis of circadian fluctuations in hepatic glucose metabolism related genes in DFAM rats (related to Fig 5).

	Control			DFAM		
Hepatic glucose metabolism related gene	*p*-value	Peak time (ZT)	Amplitude	*p*-value	Peak time (ZT)	Amplitude
GCK	0.000	22	45.388	0.000	0	54.268
PFKL	0.012	18	13.813	0.061	20	12.178
LPK	0.061	2	7.416	0.054	6	12.695
G6PC	0.037	2	31.531	0.000	6	41.945
PEPCK	0.003	8	28.313	0.002	10	25.799
TAT	0.002	16	27.469	0.943	18	6.548
