# Supplementary material for: Delayed first active-phase meal, a breakfast-skipping model, led to increased body weight and shifted the circadian oscillation of the hepatic clock and lipid metabolism-related genes in rats fed a high-fat diet
Source: PLoS One. 2018 Oct 31;13(10):e0206669. doi: 10.1371/journal.pone.0206669 (PMC6209334; doi:10.1371/journal.pone.0206669)
Supplement: S7 Table — (PDF) [file pone.0206669.s007.pdf]

Supplementary Table 7. The results of two-way ANOVA of in serum parameters, hepatic genes expression in DFAM rats.

	Two-way ANOVA		
	Time-effect	DFAM-effect	Interaction
Serum glucose	NS[a]	0.05	NS
Serum cholesterol	NS	NS	NS
Serum triglyceride	0.05	NS	NS
Serum NEFA	0.05	NS	0.05
Serum total bile acids	0.05	0.05	0.05
Serum insulin	0.05	NS	0.05
Serum corticosterone	0.05	NS	NS
BMAL1	0.05	NS	0.05
CLOCK	0.05	NS	NS
PER1	0.05	NS	0.05
PER2	0.05	NS	0.05
CRY1	0.05	NS	0.05
CRY2	0.05	NS	0.05
DEC1	0.05	NS	0.05
DEC2	0.05	NS	NS
REV-ERBα	0.05	NS	0.05
REV-ERBβ	0.05	NS	0.05
RORα	0.05	NS	NS
E4BP4	0.05	NS	0.05
DBP	0.05	NS	0.05
TEF	0.05	NS	0.05
HLF	0.05	NS	0.05

SREBP1c	0.05	NS	0.05
LXRα	0.05	0.05	NS
ACLY	0.05	NS	0.05
FAS	0.05	NS	0.05
ME1	0.05	NS	NS
PPARα	0.05	NS	NS
CPT1α	0.05	NS	0.05
ACOX1	0.05	NS	NS
HMG-CoAR	0.05	NS	NS
HMG-CoAS	0.05	NS	0.05
CYP7A1	0.05	NS	0.05
ABCG5	0.05	NS	0.05
GCK	0.05	NS	NS
PFKL	0.05	0.05	NS
LPK	0.05	NS	NS
G6PC	0.05	NS	NS
PEPCK	0.05	NS	NS
TAT	0.05	NS	NS

[a] NS, not significant ($p>0.05$); 0.05, significant ($p<0.05$)
