# Supplementary material for: Delayed first active-phase meal, a breakfast-skipping model, led to increased body weight and shifted the circadian oscillation of the hepatic clock and lipid metabolism-related genes in rats fed a high-fat diet
Source: PLoS One. 2018 Oct 31;13(10):e0206669. doi: 10.1371/journal.pone.0206669 (PMC6209334; doi:10.1371/journal.pone.0206669)
Supplement: S8 Table — (PDF) [file pone.0206669.s008.pdf]

Supplementary Table 8. The JTK_CYCLE analysis of circadian fluctuations in epididymal adipose tissue of DFAM rats (Related to S2 Fig)

	Control			DFAM		
Genes	*p*-value	Peak Time (ZT)	Amplitude	*p*-value	Peak Time (ZT)	Amplitude
BMAL1	0.000	0	38.640	0.000	0	85.835
CLOCK	0.006	20	28.602	0.054	0	19.436
PER1	0.000	12	250.198	0.000	16	138.295
PER2	0.000	14	95.984	0.000	18	119.529
CRY1	0.000	20	47.102	0.000	22	35.945
CRY2	1.000	18	14.383	1.000	10	25.548
