# Supplementary material for: Delayed first active-phase meal, a breakfast-skipping model, led to increased body weight and shifted the circadian oscillation of the hepatic clock and lipid metabolism-related genes in rats fed a high-fat diet
Source: PLoS One. 2018 Oct 31;13(10):e0206669. doi: 10.1371/journal.pone.0206669 (PMC6209334; doi:10.1371/journal.pone.0206669)
Supplement: S9 Table — (PDF) [file pone.0206669.s009.pdf]

Supplementary Table 9. The results of two-way ANOVA of gene expressions in epididymal adipose tissue of DFAM rats (Related to S2 Fig).

	Two-way ANOVA		
	Time-effect	DFAM-effect	Interaction
BMAL1	0.05	NS[a]	0.05
CLOCK	NS	0.05	NS
PER1	0.05	NS	NS
PER2	0.05	NS	0.05
CRY1	0.05	NS	NS
CRY2	NS	NS	NS

[a] NS, not significant ($p>0.05$); 0.05, significant ($p<0.05$);
