# Supplementary material for: Delayed first active-phase meal, a breakfast-skipping model, led to increased body weight and shifted the circadian oscillation of the hepatic clock and lipid metabolism-related genes in rats fed a high-fat diet
Source: PLoS One. 2018 Oct 31;13(10):e0206669. doi: 10.1371/journal.pone.0206669 (PMC6209334; doi:10.1371/journal.pone.0206669)
Supplement: S1 Fig — (PDF) [file pone.0206669.s010.pdf]

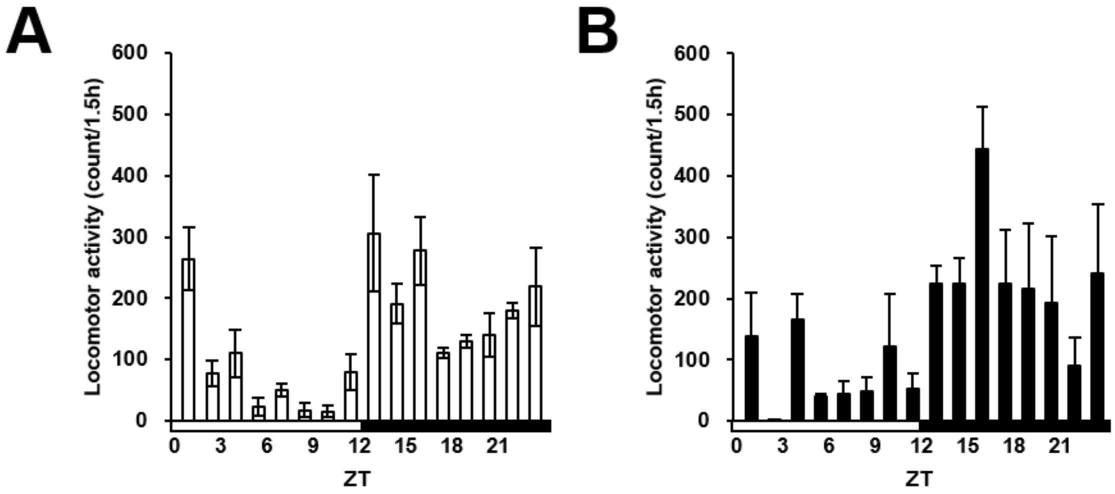

Supplementary Figure 1. DFAM did not change locomotor activity in rats fed a high-cholesterol diet. In another DFAM experiment, we monitored the locomotor activity for 24 hours. These data are the reference for the present study. Control rats were allowed access to a diet from ZT 12–24. The DFAM group was allowed access to a diet from ZT 16–4. Total activity was not different between both groups. The patterns of locomotor activity in (A) control and (B) DFAM group during a day were similar. Values showed mean ± SEM of 3 rats. The open and closed horizontal bars indicate the light (ZT 0-12) and the dark (ZT 12-24).
