# Supplementary material for: Delayed first active-phase meal, a breakfast-skipping model, led to increased body weight and shifted the circadian oscillation of the hepatic clock and lipid metabolism-related genes in rats fed a high-fat diet
Source: PLoS One. 2018 Oct 31;13(10):e0206669. doi: 10.1371/journal.pone.0206669 (PMC6209334; doi:10.1371/journal.pone.0206669)
Supplement: S2 Fig — (PDF) [file pone.0206669.s011.pdf]

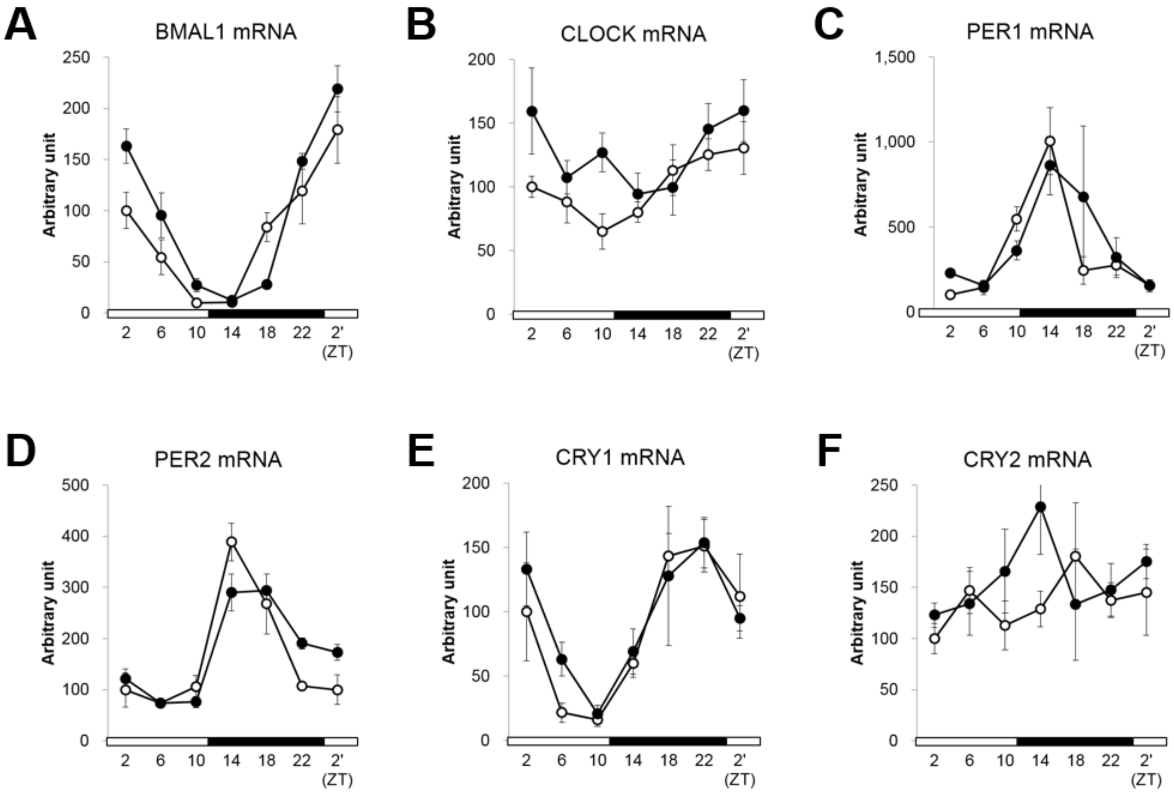

Supplementary Figure 2. DFAM slightly delayed circadian oscillation of clock genes in epididymal adipose tissue. We profiled expression of clock genes in epididymal adipose tissue. We measured circadian oscillation of mRNA of (A) BMAL1, (B) CLOCK, (C) PER1, (D) PER2, (E) CRY1, and (F) CRY2 genes in epididymal adipose tissue. The mRNA expressions of each gene were analyzed using real-time PCR and those were normalized by Apo E gene expression level. The open circle indicates control group (○) and the closed circle indicates DFAM group (●). Each value in ZT point is means ± SEM of 4 rats. The open and closed horizontal bars indicate the light (ZT 0-12) and dark (ZT 12-24), respectively. The rhythmicity of gene expressions of clock in epididymal adipose tissue were analyzed by JTK_CYCLE and the results were provided in S8 Table. The statistical significance of differences was analyzed by two-way ANOVA. The results of two-way ANOVA were described in S9 Table.
